# SPM: A history

**DOI:** 10.1016/j.neuroimage.2011.10.025

**Published:** 2012-08-15

**Authors:** John Ashburner

**Affiliations:** Wellcome Trust Centre for Neuroimaging, 12 Queen Square, London, WC1N 3BG, UK

**Keywords:** SPM, History

## Abstract

Karl Friston began the SPM project around 1991. The rest is history

## Introduction

Much has already been said about the history of SPM (Friston et al., 2007),^1^ so I have attempted to provide a slightly different perspective that will enable me to include several blatant self-citations. There will not be any equations. Instead, I will attempt to explain using the tried and tested technique of hand-waving.

This review is organised according to various releases of the SPM software from over the years. The term “SPM” does not really refer to a single piece of software, as many changes are made between each release. Major new components are usually introduced, some of which make it into prestigious journals such as NeuroImage. Mostly though, bugs are fixed, features are introduced to make life easier for users, and changes are made to ensure compatibility with whatever improvements the MathWorks make to MATLAB (in which the various incarnations of SPM were written).

When writing up a study, simply stating that SPM was used will not accurately convey how the data were actually analysed. The particular major release (SPM5, SPM8 etc., where the number refers to the approximate year) should be stated explicitly, in addition to mentioning which updates^2^ were used.

Prior to each release, there is usually a beta version of the software made available (e.g. SPM8b was the beta version of SPM8). Often, the release of a slightly unstable beta version will coincide with an SPM course, making the practical sessions a little more entertaining. Beta versions provide an opportunity for the more curious investigators to position themselves ahead of the rest of the field, by finding out about future developments and understanding new concepts. In return, the SPM developers gain feedback about what works, and what needs fixing prior to unleashing the official version.

Although some of the ancient incarnations of SPM are still available through the SPM web pages,^3^ we do not encourage their use. Mailing list^4^ queries concerning any version prior to the latest tend to be ignored by the authors. Any bugs reported have often already been fixed and other answers may not be relevant to more progressive users.

## SPM91

SPM was originally conceived by Karl Friston.^5^ It started life at the MRC Cyclotron Unit, at the Hammersmith Hospital in west London, which was a unit specialising in Positron Emission Tomography (PET). PET images were usually collected as dynamic time series, after the injection or inhalation of some positron emitting tracer. Using dynamic scans acquired from subjects who inhaled ^15^O-labelled CO_2_ or H_2_O, it was possible to invert kinetic models of the time-series to obtain physiologically meaningful estimates of model parameters, such as regional cerebral blood flow (rCBF) (Frackowiak et al., 1980). For simple models, these could be estimated from time courses at each voxel, which resulted in maps of model parameters.

These maps were originally utilised by measuring values within a handful of regions of interest (ROIs). Usually, ROIs were manually defined using the ANALYZE software package.^6^ This involved attempting to draw around brain structures in these rather noisy images, or more crudely, by placing circles around them. Regional differences among scans were computed from the average signal intensities within the various ROIs. This was a wasteful procedure, as it reduced hundreds of thousands of measurements to just a few. What was needed was an approach that was not biassed to a small number of structures, instead giving an even-handed and comprehensive assessment of differences throughout the brain.

Subtraction images were an early approach for whole brain analysis, and first used for visualising rCBF changes elicited by tasks (Lauter et al., 1985; Fox et al., 1986). A few years later, the first map of t statistics was used to localise colour processing (Lueck et al., 1989). The analysis in this work used a primitive version of the Statistical Parametric Mapping (SPM) software.

Karl wrote the original SPM in MATLAB, and it was used extensively by researchers at the Cyclotron Unit. This version is now known as SPM91, or as SPMclassic. In the spirit of open science, it was then distributed to collaborators and other interested units around the world. Within a few years, SPM had become the most popular way to analyse studies of rCBF changes.

### Image registration

The St Louis group had previously established the notion of a common anatomical or stereotactic space (Fox et al., 1988) in which to place difference maps. The issue for SPM91 was how to transform data into that space. Initially, this was done from attempts at identifying landmarks in the functional data themselves, which were subsequently used to drive the registration (Friston et al., 1989). The approach was abandoned shortly afterwards, to be replaced by a more automated, and therefore reproducible, approach (Friston et al., 1991b).

### Linear modelling

Spatial normalisation and smoothing was then followed by voxel-wise statistical analysis. At that time, the general linear model (GLM) formulation of statistical testing was not known in the community, so each type of analysis was done by a different MATLAB function.

Particular treatments, or stimuli, are likely to elicit whole patterns of signal change throughout the brain. Because the objective was to localise regionally specific effects, voxel-by voxel analyses were performed, modelling global signal changes as a confound within each analysis (Friston et al., 1990). The resulting maps were visualised via their maximum-intensity projections (known colloquially as glass-brains).

### Statistical inference

Initially, there was concern about the specificity of the inferences made, largely from outside the imaging community. Without accounting for the multitude of statistical tests, performing them at each voxel would generate an enormous rate of false-positives. A simple Bonferroni correction would be too conservative because of the spatial correlations among neighbouring voxels. What was needed was a way of predicting the rate of false positives in spatially smooth statistical maps, under the null hypothesis that there is no response anywhere in the brain.

An initial framework was developed, based on heuristics from the theory of stochastic processes and level-crossings (Friston et al., 1991a). This is when Keith Worsley enters the SPM story, as he pointed out that those heuristics were similar to established results from the theory of random fields (Worsley et al., 1992). Although several basic principles of topological inference were established at that time, Keith and his students would later contribute a number of further mathematical developments for dealing with statistical fields of various types. Until his death in 2009, Keith continued to play an important role in ensuring that things were eventually done correctly by SPM.

## SPM94

Following his development of SPM91, Karl spent a couple of years working on different topics in the USA. On his return to London, he completely re-wrote SPM and it re-emerged – lean and mean – as SPM94. The 33,500 lines of MATLAB code from SPM91 had been reduced to a mere 5700. Karl unified many of his earlier ideas into much more elegant formulations, and we now saw the emergence of the familiar push-button user interface. Along the top were three “spatial” buttons (“Realign”, “Normalize” and “Smooth”). These were followed by the “analysis” buttons (“Statistics” and “Eigenimages”) and the “results” buttons (“SPM{Z}”, “Sections” and “Render”). Along the bottom were “help” (which included a handy guide for writing a methods section of a paper, or for writing this history of SPM), “defaults” (mostly for specifying the sort of information that is now read from image headers), “images” (which displayed saggital [sic], transverse and coronal sections through the centre of an image) and “Quit”.

### Image registration

Most of the pre-processing, prior to statistical analysis, involves various forms of image registration. The approaches developed for SPM94 were described in Friston et al., (1995a), and based on an independent re-invention of Gauss–Newton optimisation.

SPM94 was the first release containing a component for motion correction (realignment). It involved using a least squares approach to estimate the six rigid-body movement parameters that align each scan to the first, which were subsequently used to re-slice the series of images.

Realigned images were then spatially transformed into the space of Talairach and Tournoux, (1988), which had become the standard in the field. As in the realignment, this involved estimating a transform that minimised the mean squared difference between an image of each subject and a template image. The procedure began by estimating the coefficients of a 12-parameter affine and nine-parameter quadratic transform, which was optionally followed by slice-wise 2D nonlinear registration. Following this estimation step, the scans were re-sampled so that they conformed to the standard space defined by the template.

After alignment, the images were spatially smoothed by convolving with a Gaussian kernel. This was typically of about 16 mm full width at half maximum.

### Linear modelling

The familiar design matrix approach for specifying linear models was introduced in SPM94, leading to much more conceptual and mathematical simplicity. The various analyses that could be performed within SPM91 had all been special cases of the GLM. Rather than focussing his effort on particular special cases, a recurring theme in Karl's work is the search for suitably generic frameworks. The flexibility of the GLM provides for great latitude in experimental design and analysis, and the adoption of factorial designs was one of the most important advances at this point.

After specifying a design matrix, the various effects were estimated according to the GLM at each and every voxel (Friston et al., 1995b) of the pre-processed data. For testing hypotheses about regionally specific condition or covariate effects, linear combinations of parameter estimates (contrasts) were constructed. The statistical tests were based on whether or not this combination was significantly greater than zero. The resulting set of test results constituted a statistical parametric map of the t statistic (known at the time as an SPM{t}).

### Statistical inference

In SPM94, the SPM{t} maps were transformed to the-unit normal distribution (SPM{Z}) and thresholded at an uncorrected level of p = 0.001. The resulting blobs were then characterised either in terms of their spatial extent or their peak height, using distributional approximations from Gaussian Field theory. The former approach is based on the probability that a blob of the observed size (or bigger) could have occurred by chance over the entire volume analysed, whereas the latter is based on the probability that the observed peak height (or higher) could have occurred by chance.

### Connectivity

In addition to the familiar statistical mapping framework, SPM94 also enabled an analysis of functional connectivity. An eigen-image analysis took a series of data, and used singular value decomposition to compute the spatial modes associated with that series, after first orthoganalising with respect various confounding effects from the design matrix. For time series data, these modes may be interpreted in terms of functional connectivity (Friston et al., 1993). Over subsequent years, the SPM software would see a number of approaches for characterising brain connectivity.

## SPM95

The potential of MRI techniques for mapping brain areas involved in cognitive processing was first demonstrated by Belliveau et al., (1991), although we cannot forget the other important contributions made that year (Stehling et al., 1991; Turner et al., 1991) and shortly afterwards (Turner, 1992; Kwong et al., 1992; Le Bihan et al., 1993). The advent of this new functional MRI (fMRI) technology led to a new wave of innovation within the field. Although a few early fMRI studies had used SPM, it was not until SPM95 that fMRI-specific developments were included.

### Image registration

In addition to the initial excitement, the advent of fMRI led to some scepticism. Motion artefacts were of particular concern (Hajnal et al., 1994), especially when head movement correlated with the stimuli presented to the subjects. Even after perfect realignment, some variance attributable to head motion remained. Karl's solution was to regress out the estimated motion parameters from the realigned data, along with their first temporal derivatives (Friston et al., 1996a). Initially, this was done as part of the motion correction procedure. A few years later, the recommended approach became one of including the estimated motion parameters as confounds during the GLM stage of the analysis.

### Linear modelling

Following motion correction, spatial normalisation and spatial smoothing, the next step in the pipeline was to fit a GLM through each voxel of the pre-processed data. Karl's early work on fMRI time series modelling established the concept that the measured fMRI signal was generated by temporally convolving the input stimuli with a linear response function (Friston et al., 1994). This response function accounted for the delay and dispersion of the haemodynamics, and originally assumed the form of a Poisson distribution. In addition, this early work set out the notion of temporal correlations, and the need for statistical tests to adjust for the effective number of degrees of freedom.

## SPM96

At around this time, Karl and a few others (including yours truly) left the Hammersmith Hospital and moved to our current location in 12 Queen Square. The group now had access to MRI, as well as PET, so we began to explore fMRI a bit more thoroughly. Shortly afterwards, we were sufficiently happy with fMRI – even for Cathy Price's language work – that we abandoned our mini-cyclotron and PET scanner. Now there was much more emphasis on developing the framework for fMRI, so a number of issues relating to fMRI analysis were revisited for SPM96. Sometimes more than once.

**Fig. 1:**
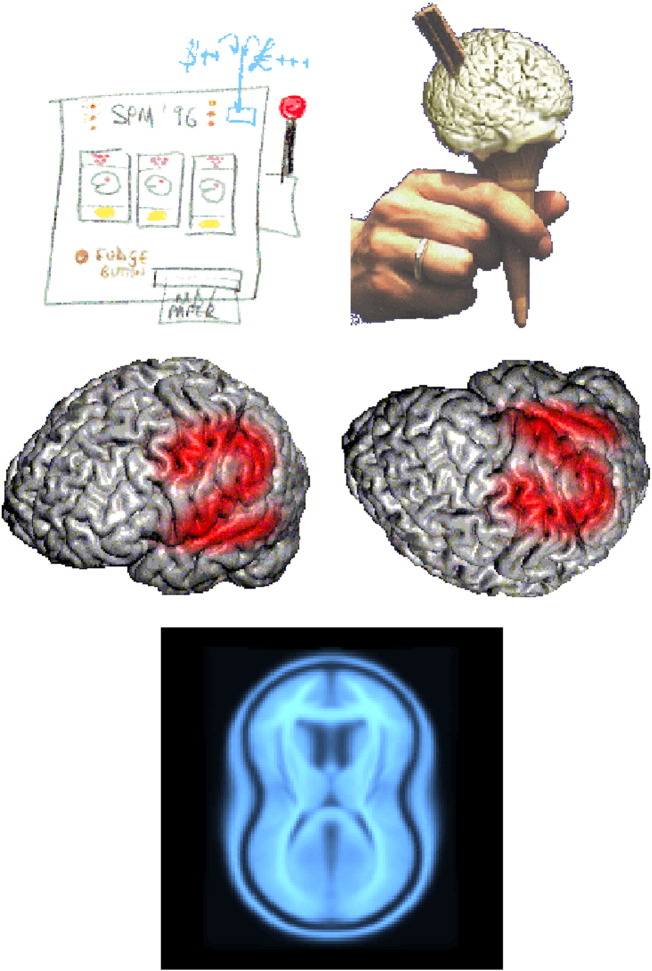
The SPM logo from over the years (96, 99, 02, 05 and 08). The 99 logo was suggested by Roger Gunn and chosen to confuse anyone unfamiliar with British culture.

Thanks to Stuart Derbyshire, SPM96 acquired a corporate identity (see Fig. 1) that reflected the ease with which neuroimagers could now analyse and publish their data.

### Image registration

Because it is the point at which data first enters the analysis stream, all image format issues need to be dealt with at the image registration step. Unlike PET, which is always collected as axial slices, MRI can be acquired in any orientation. This would cause many challenges for the local optimisation algorithms used for image registration. Also, after several questions about why disc space seemed to be filled up after registering low resolution functional data to match high resolution anatomical scans, it became evident that a solution was needed. When data were aligned within subject, the approach became one of saving the rigid-body transforms, rather than actually re-sampling the image data. This is when I introduced the “.mat” files in SPM – a step that would lead to endless confusion.

In SPM94 and SPM95, nonlinear spatial normalisation had been effected in a slice-wise manner. A fully 3D nonlinear spatial normalisation was introduced for SPM96, which eliminated some of the “swirly” effects from those earlier versions. At this time, the approach was not regularised by penalising the transformations based on some measure of deformation roughness. Instead, regularisation was achieved by limiting the number of spatial basis functions used to model displacements.

By then, the International Consortium for Brain Mapping (ICBM) had been formed with the aim of developing a probabilistic reference system for the human brain. The Montreal Neurological Institute (MNI) was part of that consortium, and they had developed a coordinate system similar to that of Talairach and Tournoux, (1988). This is when SPM switched from a Talairach-ish^7^ system to one based on spatially normalising to MNI 305 space (Evans et al., 1993). Unfortunately, MNI 305 space was not quite the same as T&T space.

### Linear modelling

The linear models of SPM96 introduced temporal smoothing of the fMRI data, such that the autocorrelations inherent to the pre-processed data were now swamped by the known autocorrelations induced by smoothing. The idea was that the effective degrees of freedom used for drawing statistical inferences could be computed from these known correlations. The original pre-colouring framework for doing this (Friston et al., 1995d) was soon superseded by the more thorough and correct formulation of Worsley and Friston, (1995).

SPM96 also introduced the possibility of modelling haemodynamic response functions using linear combinations of temporal basis functions. This allowed haemodynamic responses to vary across voxels, as well as over subjects and conditions. A few years later, the analysis of event-related experiments (Josephs et al., 1997; Friston et al., 1998) would be a useful application for the approach.

## SPM99

Developments for SPM99 continued in the direction of analysing fMRI by fitting a voxel-wise GLM through the pre-processed data, and making inferences based on frequentist statistics.

### General housekeeping

Much of the code behind SPM deals with general housekeeping, rather than interesting scientific issues. This includes reading and writing of images, user interfaces, visualisation, documentation etc. Although these aspects are generally of little scientific interest, they can have a major impact from the user's perspective. Over the years, many changes to SPM have been a direct result of commonly recurring queries on the SPM mailing list.

There are often many additional things that users would like to do, which are not part of the functionality of SPM. At around this time, we added the ability for external groups to make their own toolboxes easily callable from SPM, and provided links from our SPM web pages^8^ so that others may download them. Many of these have been very widely used.

Support for Linux and Windows platforms was introduced with SPM99. The lower cost of the PC hardware resulted in many new SPM users, and a host of new problems. Those new platforms required byte-swapping of image data to be supported, so that same data could be used on both big-endian (e.g. Sun) or little-endian (e.g. PC) computers. Initially, there were a few issues with filenames containing spaces, but these were eventually resolved. Similarly, a few crashes were caused by attempts to read from files that were opened by some other software (automated backup systems appeared to be a major cause), but I think we mostly also fixed this one.

Various problems that had arisen with large fMRI datasets required some attention, leading to the abandonment of the previous strategy of memory mapping all the scans. 32-bit computers are limited to dealing with no more than 2^32^ − 1 bytes (or sometimes 2^31^ − 1 bytes), which meant that SPM could not work with more than 4 Gbytes (or 2 Gbytes) of memory. New image I/O routines were developed to resolve this, although when working with large files, memory problems still occasionally occur in SPM as a result of the 4 Gbyte limit.

Some new, and occasionally useful, utilities were added to SPM99. In particular, the visualisation of images had been very limited in previous releases, so this received some attention.

Fitting a GLM through data is straightforward, as the solution can be found via a single matrix inversion. The situation is different for the various registration procedures used to pre-process the data. These models are nonlinear, so there is much more that can go wrong, as fitting them requires an iterative procedure to find the closest optimal solution. For this to work, the initial guess for the model parameters needs to be sufficiently close the optimum. Typically, registration works reasonably well when initial displacements are below about 3 cm and relative rotations are below about 15^o^.

MRI can be, and is, acquired in any orientation. This meant that for a substantial proportion of data, any attempt at fully automatic image registration was likely to fail. What was needed was the ability to reposition and reorient images, as well as to view the relative positions of two or more images. Additional functionality was added to the old Display button to allow orthogonal sections to be changed via button clicks. The Check Reg button was added to allow corresponding orthogonal sections through a number of images to be shown in an interactive way. It was intended for checking registration and spatial normalisation results, as well as ensuring that images were in good enough initial alignment for these routines to actually have a chance of working. One of the most common suggestions on the SPM mailing list is to try actually looking at the data via Check Reg.

### Image registration

During the period when SPM99 was being developed, there was much concern about motion artefacts within our lab. At the time, much of it was believed to be due to the way that the images were interpolated, although we later realised that the main cause is from interactions between subject head motion and the artefacts inherent in fMRI data. A number of fixes were made to the module for motion correction. These seem to have reduced the incidences of rimming artefacts, which have not been reported for a while.

A routine was added for correcting fMRI time series data for the differences in image acquisition time between slices, and based on original code by Mark D'Exposito, Geof Aquirre and Eric Zarahn. When done correctly, generative modelling involves fitting a model to the data, rather than deforming the data to fit the model. For this reason – and others – the primary SPM authors remain ambivalent about slice time correction.

Spatial normalisation involves deforming the image data from the various subjects in the study, so that they all fit to a common anatomical space. A number of improvements were made to the spatial normalisation code. Other than various important bug fixes, the main change was in the use of regularisation. By including priors on the zooms and shears used in the affine part of the registration (Ashburner et al., 1997), as well as penalising the roughness of the nonlinear deformations (Ashburner and Friston, 1999), it became possible to achieve more robust solutions. This was an early attempt to introduce some Bayesian stuff into parts SPM, although it was pretty crude at that stage.

### Linear modelling

Karl, Andrew Holmes and Jean-Baptiste Poline completely re-wrote the statistical modelling code for SPM99. This involved splitting it up into three sections: setup, estimation and results.

Analysis of a study involving a single subject may have found very significant differences, but there was no way to ensure that these would generalise to the population. It would still be a study with only *N* = 1. A challenge at that time was to devise a way of ensuring that findings could be generalised, rather than just pertaining to the subjects in the study. The issue to contend with was the fact that intra-subject (scan-to-scan) variability is not the same as inter-subject variability. Solving this required a random effects framework, which involves assuming a hierarchical observation model. Because SPM only had the machinery to do single-level (fixed-effects) analyses, a device was required to implement this. The solution was to adopt a straightforward two-pass procedure (Holmes and Friston, 1998):

• For the first pass, contrasts of parameter estimates are generated for each subject, which essentially involves computing some linear combination of the pre-processed scans (although it can more easily be done by spatially normalising a linear combination of realigned scans).
• The second level of the analysis is then simply a statistical analysis of the contrast images.

### Statistical inference

The “contrast manager” was introduced in SPM99, allowing contrasts to be specified after fitting the GLM. In addition to contrasts for t statistics, the re-written code also allowed contrast vectors (or matrices) to be specified for F tests. An F statistic is essentially a model comparison, in which a simple linear model (encoding the null hypothesis) is compared with a more complex one. Model comparison is an area that would become more important in later incarnations of SPM.

By then, Keith had extended his work on random fields to encompass, not just Z fields, but also t and F fields (Worsley et al., 1996). This meant that t maps no longer needed to be “Gaussianised” by converting to the equivalent Z statistic, and it also allowed inferences to be drawn from F maps. These developments were incorporated into SPM99.

An option for “small volume correction” was also included. If, a priori, we have a rough idea of where to expect differences, there is no need to correct for multiple comparisons throughout the whole brain. Hopefully, this feature has not been abused too much.

## SPM2

The release of SPM2 saw the beginning of the end of the frequentist era of SPM, as the software began to enter its Bayesian period of development. Although incoherent, frequentist statistics had been fashionable in science for many years. This changed with the advent of more powerful computers, meaning that it became more practical to work within the Bayesian framework.

Many components were introduced that rely on an Expectation Maximisation (EM) algorithm to estimate restricted maximum likelihood (REML) estimates of various hyper-parameters (Friston et al., 2002b). Keith Worsley first introduced us to REML, after I asked him a slightly dumb question concerning the trade-off between the matching and regularisation terms used in image registration. We first used the framework for regularising source localisation of EEG (Phillips et al., 2002), but it soon found applications in other areas. For example, deviations from i.i.d. (independent and identically distributed) assumptions can be estimated for single-level linear models or one can use this approach for fitting hierarchical observation models (Friston et al., 2002a).

This use of empirical Bayes was an important component of the paradigm shift in SPM, from frequentist inference to a Bayesian perspective. Learning priors from data depends on the conditional dependence implicit in hierarchical models, which brought the previous maximum likelihood schemes into the more general Bayesian framework.

These developments largely followed the arrival of the Gatsby Computational Neuroscience Unit in Queen Square. Many of the SPM developers became converts to the Bayesian view after attending a course on unsupervised learning at the Gatsby. Also, Andrew Holmes had recently left our group to live and work outside London, so we needed to recruit a new statistician. At the interviews, someone called Will Penny seemed to know a lot about these new Bayesian ideas that we had heard about from the Gatsby. He was hired.

An emphasis was placed on generative models for fMRI that underpinned work on biophysical modelling of haemodynamic responses and indeed the framework entailed by dynamic causal modelling (e.g., Friston et al., (2003); Penny et al., (2004)). Bayesian model selection also began to play an increasingly important role.

### General housekeeping

SPM2 saw the introduction of a crude DICOM to ANALYZE file format converter. DICOM is the extremely complicated file format that most scanner manufacturers save their data in, whereas ANALYZE was the very simple file format that we academics preferred to work with. Documentation for DICOM runs to several thousand pages,^9^ with various manufacturer-specific extensions on top of this, for which documentation can be very hard to find. Because we are not highly-paid DICOM experts, the objective was just to write a basic routine to convert a few commonly encountered flavours of DICOM, paying particular attention to those fields relating to the orientations of subjects in the scanner. Getting this part wrong would result in a lot of studies reporting findings in the wrong brain hemisphere.

In fact, a substantial number of queries on the SPM mailing list related to left-right laterality. Much of this confusion may be clarified by understanding a little more about the history of image orientations in SPM. In SPM96 and SPM99, the images entered into SPM were (mostly) assumed to be in proper ANALYZE format, which involves a left-handed coordinate system. However, the system of Talairach and Tournoux, (1988) is a right-handed coordinate system. Mapping between left- and right-handed systems requires some kind of flipping of coordinate systems. At the time, the easiest solution for SPM was to flip the image data at the spatial normalisation stage, so that un-normalised images were stored left-handed, whereas normalised images were stored right-handed. A few groups worked with right-handed coordinates throughout (and therefore did not need to flip their images).

This approach changed with SPM2. Now, instead of flipping the images, the idea was to store all images using a left-handed system, but have a “voxel-to-world” mapping that reported coordinates within a right-handed system. This would have been straightforward, except that a few labs were storing their data within a right-handed system. To accommodate this, a “flip” setting was specified in a defaults file. Those labs with a flip setting of 1 used left-handed storage, whereas those with flip set to 0 used right-handed storage. This worked – until data was shared between labs with 0 and 1 flips. At that point, chaos typically ensued.

A few years later, the NIfTI image file format emerged, which seemed to resolve many of these issues.

### Image registration

By this time, it had become accepted that much of the movement-related signal that remained in realigned fMRI data was a result of the interaction between head motion and various image artefacts. These interactions were particularly noticeable in regard to the geometric distortions and dropout – consequences of field disturbances caused by the presence of an object in the scanner. Jesper Andersson contributed a toolbox to SPM2 for correcting much of this susceptibility-by-movement interaction (Andersson et al., 2001). It also facilitated the use of field maps for achieving more accurate intra-subject alignment between functional and anatomical scans (Hutton et al., 2002).

### Linear modelling

SPM2 saw further work on dealing with data that were not i.i.d. (independent and identically distributed). The two main forms of deviation from Gaussian i.i.d assumptions that are dealt with by SPM are heteroscedasticity (different variances), and serial correlations (covariances) in fMRI. Typically, the hyper-parameters describing the variance/covariance of the pooled voxels (Friston et al., 2002b; Friston et al., 2002a) would be estimated using REML. These can then be used to pre-whiten the time series data, rendering them closer to i.i.d., thus increasing the validity of the statistical inferences. The effective degrees of freedom relies upon the Satterthwaite approximation as described in Worsley and Friston, (1995) and is commonly employed in approaches such as the Greenhouse–Geisser correction.

There is also a Bayesian perspective to this approach (Friston et al., 2002b), as it involves estimating a prior covariance model of the data. Essentially, this is estimated by partitioning the observed variance, at each and every voxel, into a voxel-specific error term and a volume-wide component generated from inter-voxel variations in the parameter estimates.

### Statistical inference

Knowledge of the prior variance allows the posterior distribution of the parameter estimates to be determined. From this, it is possible to compute the posterior probability that an estimate exceeds some specified threshold. These probabilities constitute a posterior probability map (PPM) (Friston et al., 2002a), which summarises the posterior density specific to the specified threshold. In principle, the Bayesian framework also allows inferences to be made about signal changes of some particular magnitude not occuring (where absence of evidence can mean evidence for absence).

### Connectivity

For a number of years, investigators had been identifying psychophysiological interactions (PPI), which are task-specific changes in correlations between brain regions (Friston et al., 1997). The interactions sought are actually between the task and the neural response – rather than the haemodynamic response. Therefore, a utility was introduced into SPM2 for computing the regressor for identifying PPIs, which involves deconvolving the haemodynamics from the time course, multiplying with the task regressor and finally re-convolving again (Gitelman et al., 2003).

By now, the field should have had a pretty good idea about which brain regions do what at a coarse scale. Therefore, the emphasis shifted back towards a more thorough analysis of data from a small number of pre-defined ROIs. Dynamic causal modelling (DCM) (Friston et al., 2003) was developed to enable inferences about inter-regional coupling, based on posterior probabilities. Until the recent stochastic version (Li et al., 2011), DCM assumed that all measured brain responses were evoked by the experimental design, allowing inferences about connection strengths and modulation of connection strengths by experimentally defined effects. It is based upon a generalised convolution model of how experimentally manipulated inputs evoked changes in particular cortical areas and how these changes induced changes elsewhere in the brain. The underlying model is an input-state-output dynamical model that incorporates haemodynamics. Critically, the most important parameters of these models are connection strengths among pre-selected volumes of interest.

## SPM5

Over recent years most of the development work in SPM has focussed on EEG and MEG data. The motivation was to accommodate different modalities (e.g., fMRI-EEG) within the same framework, reflecting the growing appreciation that fMRI and EEG could offer complementary information when modelled simultaneously. In particular, the poorer temporal resolution of fMRI, combined with variability of the haemodynamic response function, makes it more difficult to determine the causal directions of neural responses. At a deeper level, the focus shifted from generative models of a particular modality (e.g., convolution models for fMRI), towards models of neuronal dynamics that could explain any modality. The discovery of these integrated models would correspond to true multi-modal fusion.

Because this special issue is to mark 20 years of fMRI, I will ignore many of these EEG/MEG advances.

### General housekeeping

A number of less scientifically interesting changes were introduced for SPM5. Many of these stemmed from regular meetings of the Data Format Working Group (DFWG)^10^ in Washington, whose primary aim was to enable more straightforward exchange of data among labs. At those meetings, a number of attendees were keen to have all possible information included in the image files, so I may have made myself unpopular by pushing for very simple formats. SPM and other related software are developed by academics, who do not have the time or resources to devote to making sure that all fields are correctly filled in.

The primary result of the DFWG meetings was the NIfTI-1 data format for image data, which was incorporated into SPM5. Until then, images had been read and written in ANALYZE format, which led to a great deal of confusion about which side of the brain was which. A search through the archives of the SPM mailing list will show hundreds of questions on this subject, although with the introduction of NIfTI, the confusion seems to have been resolved. Although it is clear that the format was designed by committee, it also appears to be making (my) life much easier.

SPM5 also saw the introduction of a new user interface for some of the fMRI analysis steps. The idea was to allow easier batching, and flexibility in the order in which operations are defined. In addition to making life easier for users, much of the inspiration for the system was that it provided a framework for documenting how data were processed. This was intended to be a step towards some of the other goals of the DFWG.

Most of the code behind SPM2 had been just for user interfaces. Believe it or not, the batching system provided a much simpler framework for coding these up. Another aim was to provide documentation for each of the options, with hopefully reduced the volume of queries on the mailing list. The user interface was written such that this online help could be extracted into LaTeX files, which served as the basic framework for our beautiful SPM5 manual.

### Image registration

Previous spatial normalisation in SPM had been based on minimising the mean squared difference between the subjects' images and the template, which only works if they are acquired with much the same MRI parameters. This led to me suggesting an ad hoc workaround to Tina Good, which was published in Good et al., (2001). The popularity of these approaches led to a great deal of mailing list confusion about “customised templates”, so a new integrated spatial normalisation and segmentation routine (Ashburner and Friston, 2005) was developed for SPM5. This enabled images acquired using a wider range of MRI sequences to be spatially normalised, obviating the need to construct templates of the same image contrast. Evaluations showed it to be more accurate than the older spatial normalisation approach in SPM (Crinion et al., 2007; Klein et al., 2009).

### Statistical inference

Spatial smoothing of pre-processed data prior to statistical analysis cannot be justified from a generative modelling perspective. A more properly Bayesian approach is to include spatial smoothness priors on the model parameters Penny et al., (2005), using a Gaussian Markov Random Field. An empirical Bayesian formulation was introduced in SPM8, which determines the optimal amount of spatial regularisation, separately for each experimental effect (Penny et al., 2005). Originally, each slice was modelled separately, but in SPM8 this would be extended to be fully 3D. The SPM8 implementation would also offer other forms of spatial prior: unweighted and weighted graph-Laplacians. The unweighted graph-Laplacian prior generalises the GMRF approach, whereas the weighted version has the advantage of preserving edges. For the near future, the planned intention is to include explicit spatial basis priors (Flandin and Penny, 2007), which will include eigenvectors of the graph-Laplacian (Harrison et al., 2008). The benefit will be in the flexibility to explore a dictionary of ‘natural’ bases, providing sparse representations of neuronal responses.

## SPM8

SPM8 is the most recent major release of SPM. Again, many of the developments focussed on dynamic modelling of EEG and MEG data.

### General housekeeping

SPM8 incorporated the new batching system (*matlabbatch*) developed by Volkmar Glauche,^11^ which was derived from the system in SPM5. The main new feature concerns how it handled situations where the input to one job depends on the output of a previous job. By explicitly specifying dependencies, it becomes more straightforward to construct a single batch file describing a work flow pipeline, which can subsequently be modified easily to analyse a different data set.

Although funding had dried up for the Data Format Working Group to continue to hold face to face meetings, some members continued to hold tele-conferences to design a file format for surface-based data.^12^ The result of these discussions and emails was the GIfTI-1 file format, which – thanks to Guillaume Flandin – was subsequently supported in SPM8.

Guillaume has also developed a standalone version of SPM8, which does not require the financial expense of obtaining MATLAB licences.

### Image registration

From the queries on the SPM mailing list, it became apparent that the original unified segmentation implementation needed to be made more stable. This was achieved by modelling the whole head – rather than just the brain. In addition, the hope was that by identifying various non-brain tissue classes, a number of other useful things could be achieved. In particular, the identification of air-tissue boundaries in the head should make it possible to properly model much of the distortion artefact found in fMRI. This version was given the imaginative name of “New Segment”, although in future SPM releases it will just to be called “Segment”. Because the algorithmic changes from Ashburner and Friston, (2005) were relatively trivial, it was not written up.

Dartel (Ashburner, 2007) is a framework that was intended to achieve more accurate alignment among the brains of different subjects. An early implementation appeared as an update to SPM5, but a more finessed version was released with SPM8. Image registration essentially involves fitting a model to the data, where the model parameters encode the relative shapes of whatever the images represent. It was developed in the hope that more detailed and accurate representations of the relative shapes of brains would lead to a better understanding of the differences and similarities among various populations of subjects. In addition, aligning all scans to the population average (Ashburner and Friston, 2009) should improve the internal consistency of the approach.

More accurate alignment across subjects should also benefit fMRI studies. After spatial normalisation, one would hope that signal changes appear in similar locations in the data of all subjects in a study, which ought to reduce the need for spatial smoothing, thus allowing more precise localisation of brain function. Dartel works by aligning images of grey and white matter across subjects. The use of anatomical data to drive the alignment of functional data is limited because distortions in fMRI scans preclude accurate rigid-body alignment between functional and anatomical scans. Therefore, spatial normalisation may often be more effective for fMRI if driven by grey and white matter segmented from the fMRI themselves.

Dartel was originally intended to be an approximation of a more mathematically correct geodesic shooting approach, which models deformations via an evolving dynamical system. Since then, a Geodesic Shooting toolbox (Ashburner and Friston, 2011) was introduced in a late update of SPM8, which should be more effective in studies where the aim is to understand differences among populations (Ashburner and Klöppel, 2011). Theoretically, it achieves slightly more accurate alignment across subjects than Dartel, with more precise localisation of volumetric differences.

### Statistical inference

SPM8 included new routines for constructing Posterior Probability Maps for Bayesian model selection at the group level (Rosa et al., 2010), allowing neuroimagers to make inferences about regionally specific effects in data from a group of subjects. These comparisons are analogous to inferences using F-tests, with the advantage that the Bayesian formulation means the models do not need to be nested. The functions return between-subject posterior probability maps, created by applying the approach in a voxel-wise manner to log-model evidence data (Penny et al., 2007).

### Connectivity

DCM was further extended to use a non-linear BOLD haemodynamic response function, and also to allow the ratio of intra- and extra-vascular signals to vary in a region-specific way (Stephan et al., 2007). DCM was originally formulated using bilinear equations, where modulation of rate constants was only via the known experimental manipulations. It had been established, from invasive electro-physiological recording studies, that connections between neuronal units are influenced by activity in other units. Therefore, a nonlinear extension was developed to model such processes (Stephan et al., 2008). In addition, some timing issues, arising because fMRI slices are not acquired simultaneously, were also resolved (Kiebel et al., 2007). Adjusting the model for temporal offsets of each slice was preferable to shifting the data to fit the model.

SPM8 introduced a method for performing a random effects analysis of DCMs at the group level (Stephan et al., 2009). This involved a Bayesian model selection procedure that identifies the best model from among a set of alternatives. For example, if numerous competing hypotheses have been formulated, model selection may be used to determine which one best fits the data. Making such inferences across the population required a random-effects treatment, which is accounted for heterogeneity among the subjects and was not sensitive to outliers.

DCM is an integrated model of time series from a number of brain regions. In addition, a couple of approaches were included that deal with time series from many more voxels. A canonical variates analysis (CVA) routine was included in the results section, allowing inferences to be made about effects that are distributed in a multivariate pattern over voxels (Friston et al., 1995c; Friston et al., 1996b). There is no multiple comparison problem because all data are included in the same integrative model. The multi-variate Bayes (MVB) facility (Friston et al., 2008) was introduced to allow comparisons among various hypotheses concerning the mapping from image data to various target variables. The primary aim of MVB was not to predict brain states or classify outcomes (although this could be done), but to enable model selection among various structure-function mappings. For example, if you want to know which set of fMRI contrast images provides a more accurate characterisation of the differences between populations of subjects, then an approach such as MVB is for you.

## The future?

It is difficult to predict the exact trajectory that future development of the SPM software will follow. We anticipate that the Bayesian paradigm will continue its centrally important role, as this provides the only coherent framework for dealing with uncertainty. Similarly, hypothesis testing via Bayesian model selection will continue to bring us closer to an accurate mechanistic model of brain function.

An ongoing theme within SPM is the combined generative modelling of various types of data, such as EEG and MRI. Although more difficult to formulate, joint integrative models of data provide greater parsimony than multiple separate models of only parts of it. More parsimonious models result in fewer apparent findings, but should eventually lead to deeper understanding.

Nonlinear models are needed to parsimoniously encode the behaviour of nonlinear systems, such as the brain. We will continue to see the evolution of dynamical systems approaches, which now constitute most of the new developments.

Rather than invent solutions designed for specific problems or tasks, which are non-generalizable, and not intended to be adaptable to other purposes,^13^ the aim of the SPM developers is to discover deeper unifying principles, on which to base our modelling efforts. Unfortunately though, pragmatism often forces us into contriving short term fixes – which are much less satisfying. This will probably continue.

Over the years, SPM has grown considerably and it is no longer possible for any single developer to be familiar with all aspects. It currently contains over 400,000 lines of MATLAB code in SPM, which makes it about 70 times as large as SPM94. The discovery of unifying principles has driven much of the development of SPM, so it would be nice to find a few principles that could streamline our code and make it easier to maintain.

## Footnotes

1 See also http://www.fil.ion.ucl.ac.uk/spm/doc/history.html.

2 There are always a few bugs and changes made after each major release, which may be downloaded separately.

3 http://www.fil.ion.ucl.ac.uk/spm/.

4 http://www.jiscmail.ac.uk/spm.

5 Karl has contributed an article on the Bayesian brain to this issue, so you have my version of the history of SPM instead.

6 This is probably why most imagers now use file formats derived from that of ANALYZE.

7 I say Talairach-ish because there was no digital instantiation of a brain for actually defining the reference space. Therefore, the previous SPM template data were only approximately in T&T space.

8 http://www.fil.ion.ucl.ac.uk/spm/ext/.

9 See ftp://medical.nema.org/medical/dicom/2009/ if you don't believe me.

10 See http://nifti.nimh.nih.gov/dfwg/ for more information.

11 Although some view this arrangement as SPM8 being a toolbox for Volkmar's batching system.

12 See http://www.nitrc.org/projects/gifti/.

13 See http://en.wikipedia.org/wiki/Ad_hoc.
